# Low‐Input Assay for Transposase‐Accessible Chromatin Identifies Epigenetic Signatures of Liver Group 1 Innate Lymphoid Cells

**DOI:** 10.1002/eji.70066

**Published:** 2025-10-03

**Authors:** Kevin Schmid, Robin P. Schenk, Gabriela M. Wiedemann

**Affiliations:** ^1^ Clinical Department of Internal Medicine II, TUM School of Medicine and Health TUM University Hospital Munich Bavaria Germany

**Keywords:** ATAC, chromatin accessibility, epigenetic, ILC1, NK cell

## Abstract

Assessing chromatin accessibility in rare cell populations within tissue remains a key challenge. To address this, we present a low‐input ATAC workflow optimized for liver ILCs. The protocol is validated across cell numbers, Tn5 ratios, and library preparation steps and unveils unique epigenetic features of liver NK cells and ILC1s.

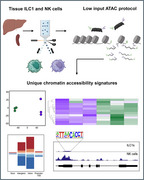

**Abbreviations**
ATAC: assay for transposase‐accessible chromatin
DAR: differential accessible region
ILC: innate lymphoid cell
NK: natural killer
TF: transcription factor

Epigenetic reprogramming is a hallmark of the innate and adaptive immune response. Rapid chromatin remodeling in response to external stimuli like infection or tissue damage ensures appropriate acute and lasting responses [1]. With regard to group 1 innate lymphoid cells (ILCs), consisting of natural killer (NK) cells and ILC1s, epigenetic programs are at the core of functional adaptations during development, activation, memory formation, and dysfunction. During development, distinct chromatin accessibility patterns assure proper functionality and gene loci of key functional genes such as *Ifng* are epigenetically primed to facilitate rapid activation [2, 3]. Moreover, NK cell and ILC1 activation, proliferation, and memory formation in response to viral infection or cytokine stimulation are fostered by epigenetic alterations, and clonal inheritance of chromatin accessibility features enables lasting NK cell memory in humans [4, 5, 6, 7]. Likewise, different homeostatic and pathologic tissue microenvironments (like the tumor microenvironment) have impact on the epigenetic landscapes and thus on functionality [8]. With regard to the central role of epigenetic programs in defining NK cell and ILC1 responses to infection and cancer as well as high inter‐organ heterogeneity, it is crucial to understand NK cell and ILC1 baseline epigenetic characteristics across different organs. However, epigenetic characterization of tissue NK cells and ILC1s is complicated by low cell numbers derived from human and mouse tissues.

Assay for transposase‐accessible chromatin followed by sequencing (ATAC‐seq), a method developed in 2013 by Buenrostro and colleagues, is a fundamental method for the assessment of chromatin accessibility and thus epigenetic state [9]. Although the initial protocol was comprehensively improved to enhance signal‐to‐noise ratio and allow for analysis of lower cell numbers [10, 11], it was not optimized for delicate tissue‐derived immune cells and their unique features such as small nuclei, high granularity, and reduced viability. Additionally, while more recent methods like single‐cell ATAC‐seq make it possible to study heterogeneous and small cell populations on a single cell basis [12], bulk ATAC‐seq provides higher sequencing depth and less noise, allowing for easier downstream analysis and identification of less pronounced regulatory elements. In order to resolve chromatin accessibility in tissue‐derived NK cells and ILC1s, we modified the omni‐ATAC protocol to a low input protocol specifically for these cell types.

First, we tested the standard protocol on 60,000 sorted splenic NK cells (Figure 1A). However, this resulted in over‐transposition and high duplication rates (Figure 1B,C), demonstrating the need to adjust the protocol. We first optimized the protocol by titrating the Tn5 reaction buffer (Figure 1B). We observed that the lowest Tn5 volume yielded a clear distribution of nucleosome‐free regions, mono‐nucleosomal regions, and di‐nucleosomal regions while maintaining perfect library concentration and average fragment size for downstream sequencing (Figure 1B). Next, we addressed the high duplication rate to ensure a sufficient depth of coverage (Figure 1C). While the initial protocol recommends using a PCR cycle number for library amplification that corresponds to 25% of the cycles required to reach maximum fluorescence during qPCR pre‐amplification, we aimed to reduce this number even further. Our results reveal that reducing the cycle count by three additional cycles does not compromise library concentration but increases average fragment size (Figure 1C).

**FIGURE 1:**
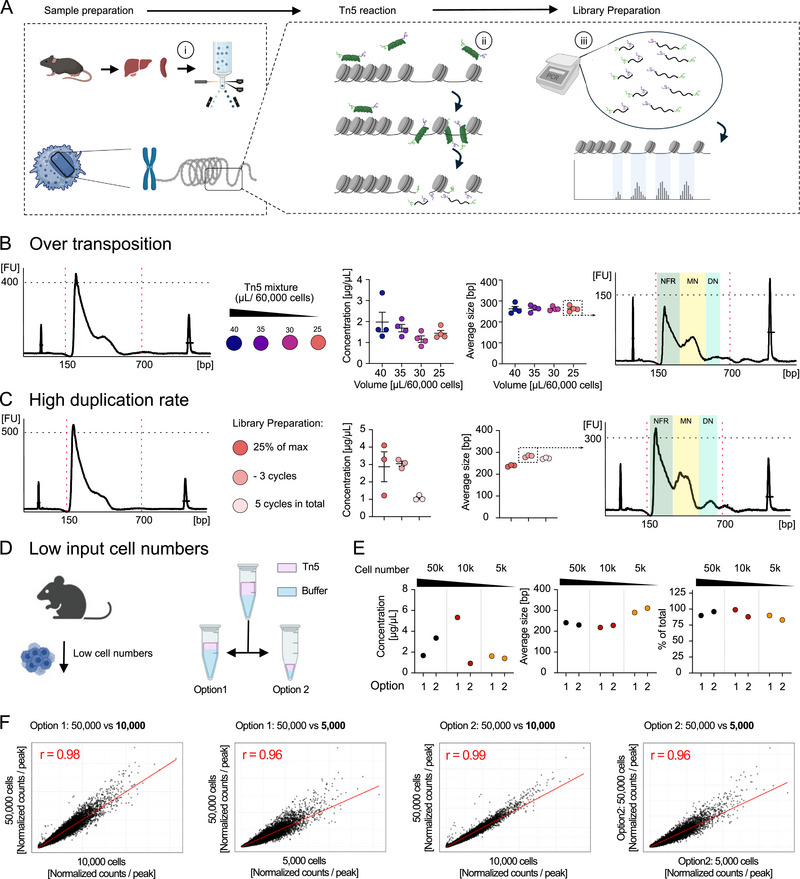
Optimization of bulk ATAC‐seq protocol for low input cell numbers. **(A)** Overview of the sample preparation process, highlighting key challenges: (i) low‐input cell numbers during initial sample preparation, (ii) over‐transposition during Tn5 reaction, and (iii) over‐amplification during library preparation. **(B)** Addressing over‐transposition: scatter plot of concentration and average fragment size after library preparation (range: 150–750 bp, extracted from BioAnalyzer; *n* = 4). Error bars represent standard error of the mean (SEM). Right side: BioAnalyzer profile of the final optimized version (25 µL reaction volume), including depiction of nucleosomal regions. **(C)** Addressing high duplication rates: scatter plot depicting concentration and average fragment size after library preparation (range: 150–750 bp, extracted from BioAnalyzer; *n* = 3). Right side: BioAnalyzer profile of the final optimized version (−3 cycles). Error bars depict SEM. **(D)** AdjustingTn5 reaction volume: Schematic of reaction setups (Option 1 and Option 2) with varying buffer volumes and constant Tn5 amount and buffer concentrations. **(E)** Scatter plot of concentration, average fragment size, and fraction of total fragments after library preparation (range: 150–750 bp, extracted from BioAnalyzer). **(F)** Pearson correlation analysis of normalized counts for all annotated peaks across different input cell numbers (Option 1 and 2: 50,000 vs. 10,000 and 50,000 vs. 5000).

Having established a suitable ATAC protocol for high numbers of primary NK cells, we proceeded to downscale input cell numbers up to 5000 cells. We compared the effects of altering the reaction buffer volume while keeping the Tn5 amount constant in two different reactions setups: option 1 and option 2. Option 2 follows the initial protocol with a down‐scaled volume based on the input cell number, while option 1 adjusts the Tn5 amount according to cell number but keeps the higher buffer volume, thus allowing easier handling (Figure 1D). To validate libraries generated with both options, we submitted the samples for sequencing. Analysis of fragment concentration, average size, and fraction within the target size range revealed neglectable differences among the two options (Figure 1E). Subsequent correlation analysis of all detected normalized peak counts demonstrated a very strong correlation between high versus lowest input cell numbers (Figure 1F). These results demonstrate that varying reaction buffer volumes still lead to robust fragment distribution as long as Tn5 amount is adjusted to input cell number. In summary, we have developed an easy‐to‐handle and reliable method for low‐input bulk ATAC of primary NK cells, which provides comparable results in low‐ versus high‐input cell numbers.

Next, we proceeded to analyze epigenetic features of liver Group 1 ILCs. To this end, we sorted NK cells and ILC1s from mouse livers and performed our optimized ATAC protocol. Principal component analysis (PCA) of all detected peaks revealed a clear separation between liver NK cells and liver ILC1s along PC1 (Figure 2A). In total, we identified 30,129 differentially accessible regions (DARs, *p*_adj_ < 0.05, |fold change| > 1.5) between the two groups (Figure 2B). Stratification by annotated genomic regions revealed that most DARs between ILC1s and NK cells are found in intergenic and intronic putative enhancer regions (Figure 2C). Transcription factor (TF) motif discovery analysis revealed Ets1 and interferon regulatory factor (IRF) motifs among top enriched motifs in liver NK cells, featuring key TFs for NK cell development and function [13] (Figure 2D). By contrast, liver ILC1 DARs were enriched for CTCF motifs, a TF involved in 3D chromatin organization, as well as NRF1, a key regulator of mitochondrial biogenesis with currently unexplored role in ILCs [14]. Gene set enrichment analysis revealed enrichment of cytokine and lymphocyte differentiation pathways in ILC1‐specific open chromatin regions (Figure S1A). Analysis of the *Eomes* and *Cxcr6* loci (Figure 2E,F) and other loci of genes highly expressed in NK cells or liver ILC1s revealed cell type–specific chromatin accessibility patterns mirroring typical gene expression profiles [15] (Figure 2G, Figure S1B). Within the accessible regions, we identified binding sites for TFs known to regulate ILC1 and NK cell function (Figure 2H). Overall, our biocomputational analysis confirms quality and biological relevance of our ATAC data and reveals differential epigenetic states in liver ILC1s versus NK cells.

**FIGURE 2:**
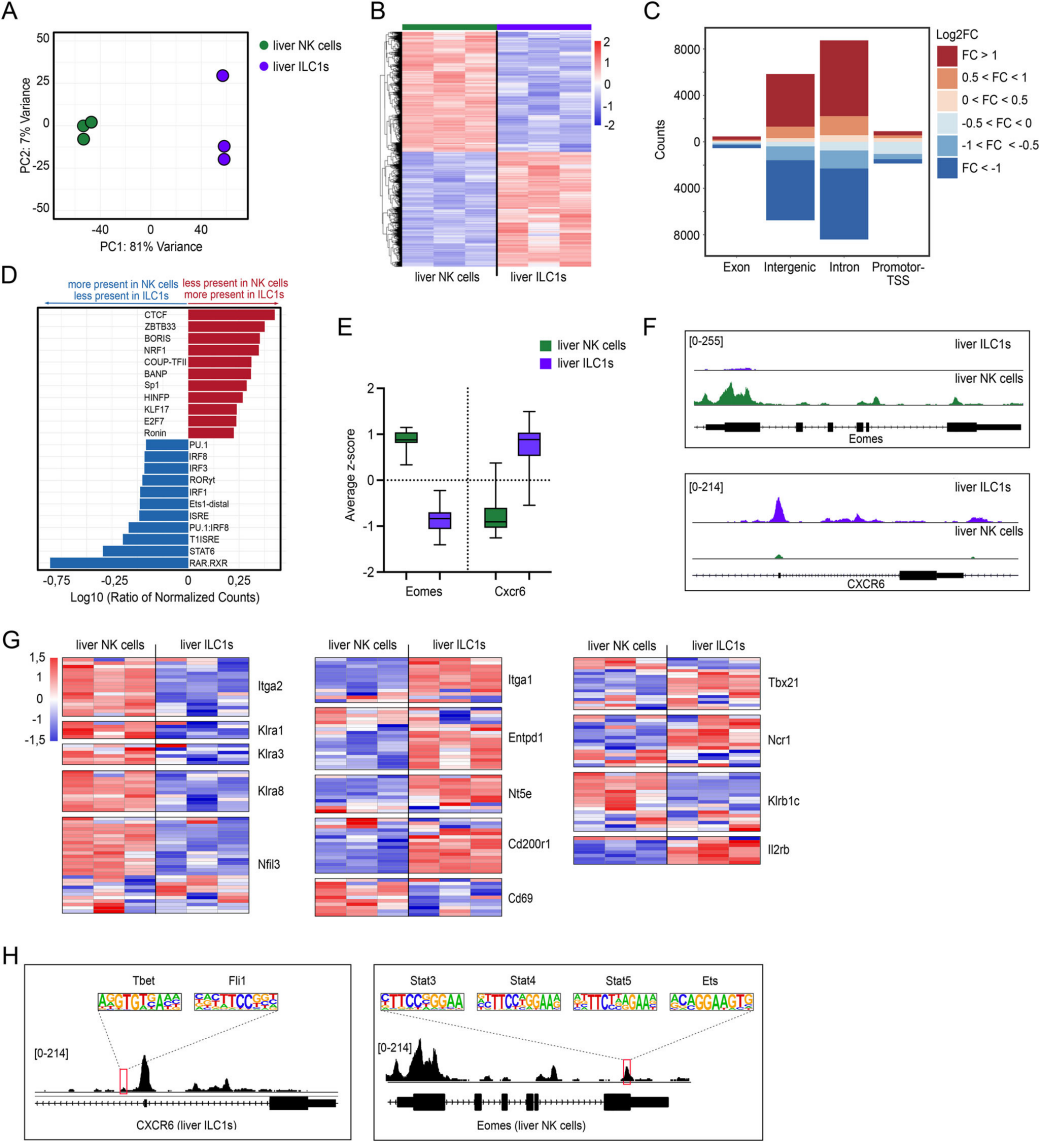
Analysis of bulk ATAC‐seq data from primary liver group 1 ILCs. NK cells and ILC1s were isolated from healthy C57BL/6 livers and sorted by fluorescent‐activated cell sorting (*n* = 3). ATAC was performed on fresh cells according to protocol. **(A)** PCA of detected peaks (107,945 features). **(B)** Heatmap analysis of 30,129 DARs between ILC1s and NK cells (|FC| > 1.5, *p*_adj_ < 0.05). **(C)** Stacked bar graph of significant annotated regions (*p*_adj_ < 0.05), categorized by accessibility. **(D)** Bar plot showing the top 11 log_10_‐transformed ratios of normalized counts for TF motifs classified as more and less accessible. **(E)** Box plot showing *z*‐scores of all DARs annotated to *Eomes*, *Cxcr6*, with whiskers at 0.05 and 0.95 percentiles. Box represents SEM. **(F)** Gene tracks of the *Eomes* and *Cxcr6* loci. **(G)** Heatmap displaying gene accessibility in detected peaks across selected gene regions (*p*_adj_ < 0.05). **(H)** Depiction of selected TF motifs identified within the respective gene loci.

In summary, we provide an optimized and reproducible low input ATAC protocol adjusted to tissue ILCs. By enabling reliable epigenetic profiling from limited cell numbers, this protocol offers new opportunities to investigate epigenetic properties of rare ILC populations in tissues at steady state and in response to perturbations such as infection, tissue damage, or malignancy. We believe that this method will be broadly applied and significantly contribute to a deeper understanding of tissue immunity.

## Conflicts of Interest

The authors declare no conflicts of interest.

## Supporting information




**Supporting File** eji70066‐sup‐0001‐SuppMat.pdf.

## Data Availability

The data that support the findings of this study is openly available at the GEO repository under GEO accession number GSE296068.
