# Supplementary material for: Low‐Input Assay for Transposase‐Accessible Chromatin Identifies Epigenetic Signatures of Liver Group 1 Innate Lymphoid Cells
Source: Eur J Immunol. 2025 Oct 3;55(10):e70066. doi: 10.1002/eji.70066 (PMC12493264; doi:10.1002/eji.70066)
Supplement: Supplementary file 1 — Supporting File eji70066‐sup‐0001‐SuppMat.pdf. [file EJI-55-e70066-s001.pdf]

Low Input Assay for Transposase-Accessible Chromatin Identifies Epigenetic Signatures of Liver Innate Lymphoid Cells Type 1 versus Natural Killer Cells

Kevin Schmid[1], Robin P. Schenk[1], Gabriela M. Wiedemann[1]

[1] TUM School of Medicine and Health, Department of Clinical Medicine – Clinical Department for Internal Medicine II, University Medical Center, Technical University of Munich, Germany

Corresponding author:
Kevin Schmid
Department for Internal Medicine II
University Medical Center, Technical University of Munich
Ismaninger Str. 22, 81675 Munich
Germany
E-Mail: kevin.schmid@tum.de

Keywords: Epigenetic, Chromatin Accessibility, ATAC, NK cell, ILC1

Material and Methods

Mice

C57BL/6J mice were bred and housed under specific pathogen-free conditions at a regular 12 h light–dark cycle with access to food and water ad libitum. All animal experiments were approved by the District Government of Upper Bavaria (Vet 02-21-118) and performed in compliance with the German Animal Welfare and Ethical Guidelines of the TUM Hospital, Technical University of Munich, Germany.

Isolation and Enrichment of Liver Lymphocytes

Liver tissue was harvested from C57BL/6J mice (24 weeks old, male) under sterile conditions. After mechanical disruption, liver tissue was enzymatically digested in Collagenase IV [0.5 mg/mL] and DNase [10 µg/mL]) to dissociate lymphocytes from parenchymal tissue. The cell suspension was passed through a 70 µm cell strainer to remove debris. Using a Percol® gradient (550 G, 20°C, 20 minutes, acceleration 9, deceleration 1), lymphocytes were separated from hepatocytes and erythrocytes. The interphase was collected and washed once with PBS before the cells were further enriched using Miltenyi MACS kit (#130-115-818).

Staining and sorting

Enriched lymphocytes were stained for fluorescence-activated cell sorting (FACS) for 20 minutes on ice with anti-CD3 (#100222), anti-CD19 (#115530), anti-F4/80 (#123118), anti-NK1.1 (#108728), anti-CD49b (#108906), and anti-NKp46 (#142606) and Fixable Viability Dye (#65-0866-14). Sterile cell sorting was performed using a BD FACSAria™ Fusion III device. NK cells were defined as $CD3^{neg}$ $CD19^{neg}$ $F4/80^{neg}$ $NK1.1^{pos}$, $CD49a^{neg}$ $CD49b^{neg}$. ILC1s were defined as $CD3^{neg}$ $CD19^{neg}$ $F4/80^{neg}$ $NK1.1^{pos}$, $CD49a^{pos}$ $CD49b^{neg}$. Cells were collected into low-bind Eppendorf tubes containing high FCS FACS buffer (10%). After sorting, the cells were processed for ATAC-seq.

Assay for Transposase-Accessible Chromatin

**Resuspension Buffer (RSB)**	
1M Tris-HCL pH 7.4	500 µL
4M NaCl	125 µL
1M $MgCl_2$	150 µL
DEPEC-treated H_2O	49,225 mL

**2x Tagment DNA Buffer (TDB)**	
1M Tris-HCL pH 7.6	200 µL
1M $MgCl_2$	100 µL
Dimethyl Formamide	2 mL

RSB 1+2 – make fresh		
	**RSB1: 50 µL/ sample**	**RSB2: 1 mL/ sample**
RSB	48,75 µL	990 µL
10% NP40	0,5 µL	-
10% Tween-20	0,5 µL	10 µL
2% Digitonin	0,25 µL	-

**Transposition Mixture**	
	25 µL/ sample [µL]
2x TD buffer	12,5
Transposase enzyme	1,25
PBS	8,25
2% digitonin	0,125
10% Tween-20	0,25
H_2O	2,625

The protocol was adapted from Corces et al. (Corces et al., 2017) and modified as followed: fresh sorted cells were pelleted at 600 rcf for 5 minutes at 4 °C. After discarding the supernatant, the pellet was resuspended in 50 µL of ice-cold RSB1 and pipetted up and down three times. The cell suspension was kept on ice for 3 minutes before RSB1 was washed out with 1 mL of RSB2, followed by gentle inversion of the tube. Nuclei were then pelleted at 600 rcf for 10 minutes, and the resulting pellet was resuspended in an appropriate volume of Tn5 mixture. For 60,000 Group 1 ILCs, 25 µL was used. After a 30-minute digestion at 37 °C with agitation at 1000 rpm, DNA was isolated and purified using the Qiagen MinElute Kit (#28004). In a subsequent step, the fragmented DNA was pre-amplified, and a small portion of the sample was used for qPCR to determine the number of additional cycles required for library preparation. The library was generated according to Buenrostro et al. (Buenrostro, Giresi, Zaba, Chang, & Greenleaf, 2013).

PCR Amplification	
Transposed DNA	10 µL
DEPEC-treated H_2O	10 µL
25M Primer 1	2,5 µL
25M Primer 2	2,5 µL
2x PCR Master Mix	25 µL

Cycling Condition (6 cycles)	
5 min	72°C
30 sec	98°C
30 sec	98°C
10 sec	63°C
60 sec	72°C

qPCR side reaction	
Pre-amplified DNA	5 µL
DEPEC-treated H_2O	2 µL
25M Primer 1	0,25 µL
25M Primer 2	0,25 µL
2x SYBR Green Master Mix	7,5 µL

Cycling Condition (19 cycles)	
10 min	95°C
10 sec	98°C
30 sec	63°C
60 sec	72°C

To enrich DNA fragments within the desired size range and to avoid sequencing primers, we performed an additional size-selection step using AMPure beads. First, 0.6× of the initial bead volume was added to the sample to remove large fragments (>700 bp). Next, an additional 2.68× bead volume was added to the transferred supernatant get rid of small DNA fragments (primers (<100 bp)). The beads were then washed twice with 80% ethanol, air-dried, and resuspended in DEPC-treated water. DNA concentration was measured using a Qubit fluorometer, and library size was assessed using an Agilent DNA High Sensitivity Chip. Bioanalyzer profile was quantified using Agilent 2100 Expert Software B.02.12 (SR2).

Bioinformatic Analysis

For further computational analysis the raw sequencing files were trimmed for adapter contamination and low quality bases with Trimmomatic 0.39 (Bolger, Lohse, & Usadel, 2014) and aligned against the *Mus musculus* genome assembly GRCm38.p6 using Bowtie2 2.5.0 (Langmead & Salzberg, 2012). Post-alignment, mitochondrial and low quality fragments were removed using pysam 0.22.0 (Bonfield et al., 2021; Li et al., 2009). Fragment duplicates were filtered out via Picard 3.1.1 and alignments to regions defined in the ENCODE blacklist (Amemiya, Kundaje, & Boyle, 2019) were removed. Broad and narrow peaks were called for each sample separately using MACS 3.0.1 (Zhang et al., 2008) in BAMPE mode. All identified overlapping peaks were subsequently merged with BEDtools 2.30.0 (Quinlan & Hall, 2010) to form a set of non-overlapping fragment pile-up regions that were annotated based on their nearest transcription start site with HOMER 5.1 (Heinz et al., 2010) before counting sample specific fragment occurrences per individual region using featureCounts 2.0.6 (Liao, Smyth, & Shi, 2013). Differences in chromatin accessibility between sample conditions were then assessed with DESeq2 1.38.3 (Love, Huber, & Anders, 2014).

Visulatization packages

All analyses were conducted using R version 2023.09.0+463. Correlation analysis was performed using Persons corrleation. Principal Component Analysus (PCA) was corried out utilizing the DESeq2 package (1.42.1) (Love et al., 2014). Heatmaps were generated using

pheatmap (1.0.12). Genset Enrichment analysis (GSEA was conducted using the clusterProfiler (4.10.1), msigdbr (7.5.1), enrichplot (1.22.0) and DOSE (3.28.2) packages. Genomic data visulatisaiton was carried out using IGV viewer (version 2.11.2-2). Additional data visualizations were made with ggplot2 (3.5.0).

Statistical analysis and figure generation

Data analysis was perfomed using GraphPad Prism 10.0. Figures were created using BioRender.

Literature (Material and Methods)

Amemiya, H. M., Kundaje, A., & Boyle, A. P. (2019). The ENCODE Blacklist: Identification of Problematic Regions of the Genome. *Scientific Reports, 9*(1), 9354. doi:10.1038/s41598-019-45839-z

Bolger, A. M., Lohse, M., & Usadel, B. (2014). Trimmomatic: a flexible trimmer for Illumina sequence data. *Bioinformatics, 30*(15), 2114-2120. doi:10.1093/bioinformatics/btu170

Bonfield, J. K., Marshall, J., Danecek, P., Li, H., Ohan, V., Whitwham, A., . . . Davies, R. M. (2021). HTSlib: C library for reading/writing high-throughput sequencing data. *GigaScience, 10*(2). doi:10.1093/gigascience/giab007

Buenrostro, J. D., Giresi, P. G., Zaba, L. C., Chang, H. Y., & Greenleaf, W. J. (2013). Transposition of native chromatin for fast and sensitive epigenomic profiling of open chromatin, DNA-binding proteins and nucleosome position. *Nature Methods, 10*(12), 1213-1218. doi:10.1038/nmeth.2688

Corces, M. R., Trevino, A. E., Hamilton, E. G., Greenside, P. G., Sinnott-Armstrong, N. A., Vesuna, S., . . . Chang, H. Y. (2017). An improved ATAC-seq protocol reduces background and enables interrogation of frozen tissues. *Nature Methods, 14*(10), 959-962. doi:10.1038/nmeth.4396

Heinz, S., Benner, C., Spann, N., Bertolino, E., Lin, Y. C., Laslo, P., . . . Glass, C. K. (2010). Simple Combinations of Lineage-Determining Transcription Factors Prime cis-Regulatory Elements Required for Macrophage and B Cell Identities. *Molecular Cell, 38*(4), 576-589. doi:https://doi.org/10.1016/j.molcel.2010.05.004

Langmead, B., & Salzberg, S. L. (2012). Fast gapped-read alignment with Bowtie 2. *Nature Methods, 9*(4), 357-359. doi:10.1038/nmeth.1923

Li, H., Handsaker, B., Wysoker, A., Fennell, T., Ruan, J., Homer, N., . . . Subgroup, G. P. D. P. (2009). The Sequence Alignment/Map format and SAMtools. *Bioinformatics, 25*(16), 2078-2079. doi:10.1093/bioinformatics/btp352

Liao, Y., Smyth, G. K., & Shi, W. (2013). featureCounts: an efficient general purpose program for assigning sequence reads to genomic features. *Bioinformatics, 30*(7), 923-930. doi:10.1093/bioinformatics/btt656

Love, M. I., Huber, W., & Anders, S. (2014). Moderated estimation of fold change and dispersion for RNA-seq data with DESeq2. *Genome Biology, 15*(12), 550. doi:10.1186/s13059-014-0550-8

Quinlan, A. R., & Hall, I. M. (2010). BEDTools: a flexible suite of utilities for comparing genomic features. *Bioinformatics, 26*(6), 841-842. doi:10.1093/bioinformatics/btq033

Zhang, Y., Liu, T., Meyer, C. A., Eeckhoute, J., Johnson, D. S., Bernstein, B. E., . . . Liu, X. S. (2008). Model-based Analysis of ChIP-Seq (MACS). *Genome Biology, 9*(9), R137. doi:10.1186/gb-2008-9-9-r137

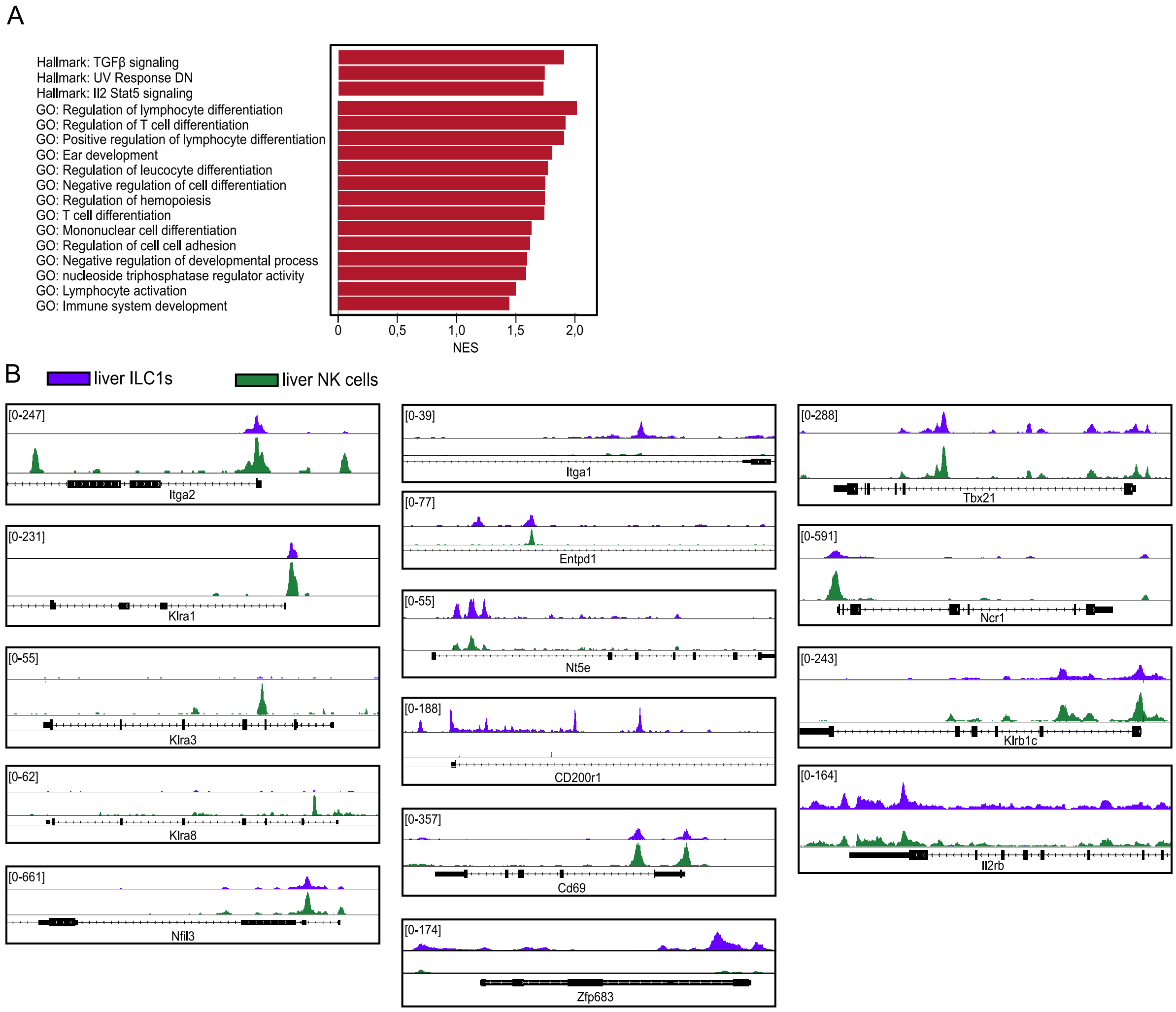

Figure Legend Supplementary Figure:

Figure S1. Gene set enrichment analysis (GSEA) and gene tracks of selected genes **(A)** GSEA of Hallmark and Gene Ontology (GO) gene sets was performed using the maximum |stat| values of DARs between ILC1s and NK cells with FC > 0 and padj < 0.05. **(B)** Gene tracks of selected gene regions, shown as auto-scaled grouped tracks.
